# Prevalence of Non Alcoholic Steatohepatitis (NASH) and association with body mass index in obese adolescents in Padang

**DOI:** 10.1186/1687-9856-2013-S1-P89

**Published:** 2013-10-03

**Authors:** Lita Farlina, Eka Agustia Rini, Rahmi Lestari, Susetyo Cahyohadi, Asviandri, ES Zul Febrianti

**Affiliations:** 1 Endocrinology Division Department of Child Health, Medicine Faculty, Andalas University/Dr. M.Djamil Hospital Padang

**Keywords:** NASH, BMI, obese adolescents

## Background

The prevalence of obesity in children increased from year to year and cause a variety of chronic diseases such as Non Alcoholic Steatohepatitis (NASH). NASH is a liver inflammation due to accumulation of fat in the liver, which had no relationship with alcohol consumption, but associated with obesity and diabetes factors. In most cases, diagnosis of NASH was established by elevated levels of aminotransferase enzymes.

## Objective

To determine the prevalence of NASH and association with body mass index (BMI) in obese adolescents in Padang.

## Methods

Cross sectional study was conducted in 80 obese adolescents which is comprised of a study of obese adolescents in 2010 with a population of obese students with anthropometric (bodyweight, bodyheight, BMI) and aminotransferase enzymes measurement. All data are presented as means±SD. Statistical analyses were conducted using t and Mann Whitney U tests. P<0.05 was considered significant.

## Results

Mean age was16 years 4 months, body weight 84.61±11.88 kg and body height 1.62±0.08 m. Mean of AST was 30.6 U/L±24.089, ALT 43.1 U/L±11.83 and ratio of AST/ALT 1.05±0.68. High levels of ALT (>40 U/L) was found in 27 cases (33%). Subject with high level of ALT had a higher BMI values compared with normal ALT (p<0.05).

## Conclusion

The prevalence of NASH in obese adolescents was 33%. There’s a significant association between elevated of BMI in obese adolescents with NASH.

